# Extracting research-quality phenotypes from electronic health records to support precision medicine

**DOI:** 10.1186/s13073-015-0166-y

**Published:** 2015-04-30

**Authors:** Wei-Qi Wei, Joshua C Denny

**Affiliations:** Department of Biomedical Informatics, Vanderbilt University, Nashville, TN 37203 USA; Department of Medicine, Vanderbilt University, Nashville, TN 37203 USA

## Abstract

The convergence of two rapidly developing technologies - high-throughput genotyping and electronic health records (EHRs) - gives scientists an unprecedented opportunity to utilize routine healthcare data to accelerate genomic discovery. Institutions and healthcare systems have been building EHR-linked DNA biobanks to enable such a vision. However, the precise extraction of detailed disease and drug-response phenotype information hidden in EHRs is not an easy task. EHR-based studies have successfully replicated known associations, made new discoveries for diseases and drug response traits, rapidly contributed cases and controls to large meta-analyses, and demonstrated the potential of EHRs for broad-based phenome-wide association studies. In this review, we summarize the advantages and challenges of repurposing EHR data for genetic research. We also highlight recent notable studies and novel approaches to provide an overview of advanced EHR-based phenotyping.

**Electronic supplementary material:**

The online version of this article (doi:10.1186/s13073-015-0166-y) contains supplementary material, which is available to authorized users.

## Introduction

The dramatic rise of inexpensive and dense sequencing technologies over the past decade has led to many genetic discoveries. Since the completion of the Human Genome Project in 2003, genome-wide association studies (GWASs) alone have markedly accelerated our search for genetic influences on diseases [1], resulting in the identification of more than 10,000 single nucleotide polymorphisms (SNPs) associated with over 250 different phenotypes [2]. These phenotypes include specific diseases (for example, breast cancer or rheumatoid arthritis) and observable traits (for example, height, skin pigmentation or drug response). Similarly, more recent efforts to look at rare variants through next-generation sequencing technologies have identified causative SNPs for rare diseases [3] as well as important modulators for some common diseases [4-6]. Through these efforts, genetic determinants of many human diseases and, more recently, therapeutic responses, are being deciphered.

Traditionally, genetic studies have leveraged purpose-built cohorts [7,8] (such as the Wellcome Trust Consortium [9], Framingham Heart Study [10] and Human Heredity and Health in Africa Consortium [11]). These studies often use self-report questionnaires and/or clinical staff to obtain participant phenotypes. While this approach provides quality phenotypes and high repeatability in the assessment of given traits, considerable challenges remain [12,13], such as slow patient accrual [14], inadequate sample size [15,16] and high cost [17]. As genotyping and sequencing costs have significantly decreased [18-20] and computing power has increased, the lack of large cohorts with adequately defined phenotypes has hindered discovery of genetic factors influencing disease [21].

In recent years, the growth of electronic health records (EHRs) has been recognized as a viable and efficient model for genetic research. In this review, we summarize the advantages and challenges of repurposing EHR data for genetic research and highlight significant initiatives, notable studies and novel approaches. Accumulated successes have demonstrated that EHRs contain rich information and hold promise for establishing more detailed phenotypes in future.

## Combining electronic health record phenotypes and genetic data

The recent widespread adoption of EHRs in the United States represents an unprecedented opportunity to leverage clinical data generated as a byproduct of healthcare for genetic discovery. An EHR system is primarily designed for routine clinical care. Early studies of EHRs focused on the challenge of their implementation [22-26] and investigated their direct benefits for patient care, including quality improvement, cost savings and interoperability [27-33]. Beginning in the 1990s, several institutions began collecting DNA samples from volunteer patients and depositing them in biobanks (Table 1). DNA samples are often accrued from leftover biospecimens collected for routine clinical testing. Many of them can be linked to individual EHRs that have been scrubbed of identifying information. These EHR-linked DNA biobanks have the potential to propel the discovery of the genetics underlying clinical phenotypes [34,35].

**Table 1:** **Efforts and incentives to leverage clinical data for genomics research**

**Projects**	**Region**	**Start year**	**Website**	**Aims**
eMERGE	United States	2007	http://emerge-network.org [152]	To develop methods and best practices for the utilization of EHRs for genetic research
i2b2	United States	2004	http://www.i2b2.org [153]	To provide researchers with useful tools to leverage EHRs for clinical and genetic research
PGPop	United States	2010	http://pgpop.mc.vanderbilt.edu [59]	To understand how a person’s genes affect his or her response to medicines
deCODE genetics	Iceland	1996	http://www.decode.com [60]	To leverage population-based and EHR-linked biosamples to investigate inherited causes of common diseases
UK Biobank	United Kingdom	2007	http://www.ukbiobank.ac.uk [61]	To improve the prevention, diagnosis and treatment of a wide range of serious and life-threatening illnesses through a collection of around 500,000 volunteers' biosamples and clinical information
MVP	United States	2011	http://www.research.va.gov/mvp [52]	To enroll one million volunteers and use their clinical and genetic data to improve health care for veterans
KP RPGEH	United States	2009	http://www.rpgeh.kaiser.org [53]	To examine the genetic and environmental factors that influence common diseases
CKB	China	2004	http://www.ckbiobank.org [154]	To explore the complex interplay between genes and environmental factors on the risks of common chronic diseases

CKB, China Kadoorie Biobank; eMERGE, The Electronic Medical Records and Genomics Network; i2b2, Informatics for Integrating Biology and the Bedside; KP, Kaiser Permanente; MVP, Million Veteran Program; PGPop, Pharmacogenomic Discovery and Replication in Very Large Patient Populations; RPGEH, Research Program on Genes, Environment, and Health.

EHRs contain a wealth of clinical information, but this information is not always in readily minable formats. Designed for clinical care, diagnoses may only be mentioned in clinical notes, and billed diagnoses may later be rejected as the physician learns more. Thus, to identify populations with high accuracy takes careful thought and domain knowledge.

**Figure 1:**
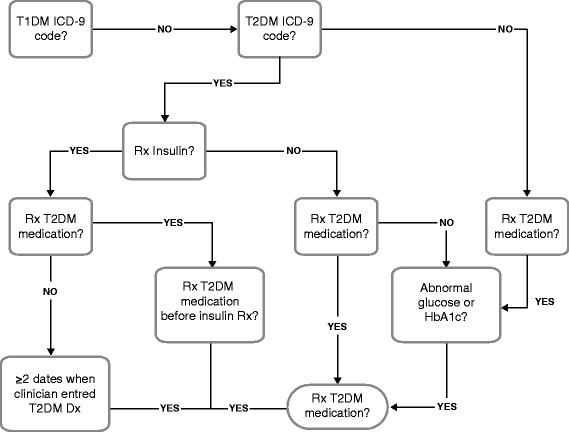
Algorithm for the identification of subjects with type 2 diabetes. Normal glucose values are random glucose >200 mg/dl, fasting glucose >125 mg/dl. Normal HbA1c ≥6.5%. Dx, diagnosis; HbA1c, hemoglobin A1c; ICD-9, International Classification of Diseases, Ninth Revision; Rx, treatment; T1DM, type 1 diabetes mellitus; T2DM, type 2 diabetes mellitus. Figure reprinted with permission from Kho *et al.* [57].

Leveraging EHRs for phenotyping generally involves collaboration across disciplines. Typically, domain experts work with clinical informaticians to create and execute an algorithm to query the EHR for subjects with the target phenotype and randomly select cases for review. Both domain experts and clinical informaticians are irreplaceable during the process. Domain experts understand the target phenotype and its representation in EHRs, while clinical informaticians know where and how to extract corresponding information. Validation is another important part of the process that not only measures an algorithm’s performance but also enhances its capability for inter-institutional sharing [36]. An algorithm may be revised and validated iteratively until its performance achieves a desired goal. An example phenotype algorithm is presented in Figure 1.

**Figure 2:**
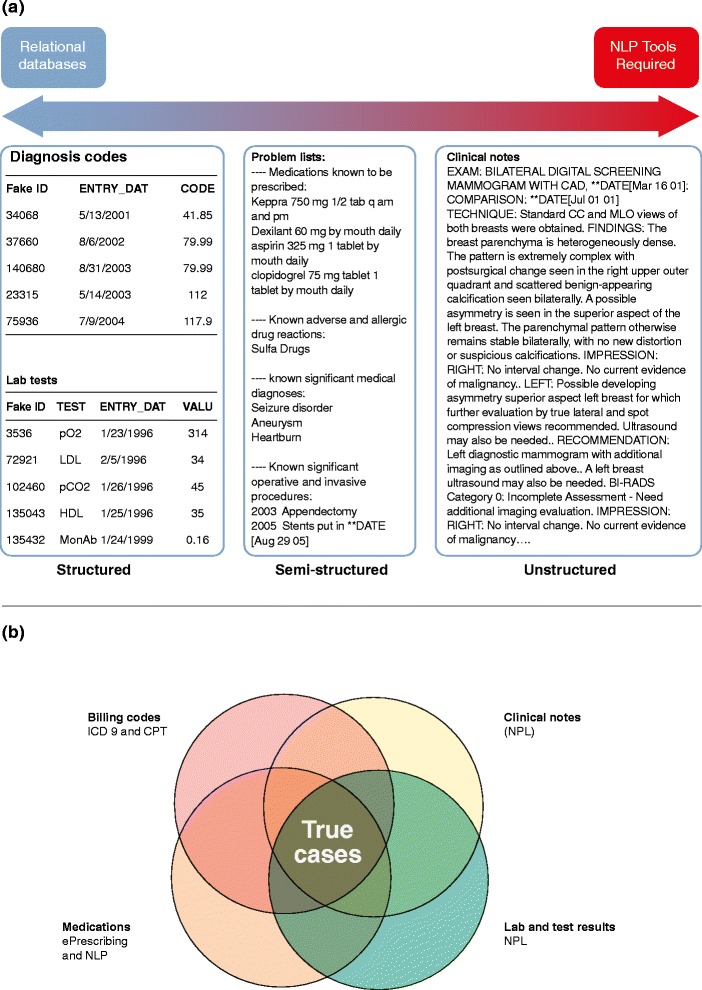
EHR data structure and accurate phenotyping. **(a)** Electronic health record (EHR) data can be structured or unstructured. Structured data are easy to retrieve whereas unstructured data require additional tools to be used for phenotyping, such as natural language processing (NLP). **(b)** Accurate phenotyping often requires extracting information from billing codes, prescriptions, laboratory tests and clinical notes. This information can be either structured or unstructured. ICD-9, International Classification of Diseases, Ninth Revision.

EHR data come in both structured and unstructured formats (Figure 2a), and the use of both types of information can be essential for creating accurate phenotypes (Figure 2b). Billing codes (for both diagnosis and procedures), laboratory test results, and growing amounts of prescription data are in structured formats that are easily stored in relational databases for rapid and straightforward retrieval [37]. Using natural language processing (NLP) pipelines and text mining techniques to scan narrative data for pertinent keywords has greatly expanded the usefulness of EHRs for research purposes. Furthermore, the presence of textual, narrative information in the form of clinical notes allows researchers to review given cases for validation of a phenotype algorithm or for careful evaluation of obscure phenotypes that may not be clearly or consistently recorded in billing code data, such as specific drug adverse events or rare diseases.

## Advantages of electronic health records for genomic medicine

EHRs have several distinct advantages for genetic research, including cost efficiency, the large amounts of available clinical data, and the ability to analyze data over time.

Early GWASs used relatively small sample sizes primarily because of the significant costs of genotyping and patient accrual. More recent studies have combined many separate GWASs via meta-analyses to yield populations of up to hundreds of thousands of patients [38]. In these cases, GWAS data are reused, but their reuse may be limited to the phenotypes already collected or require patient re-contact, which can be costly. With EHR-linked genetic data, researchers can reuse patient data for many diverse studies [39]. Thus, the marginal cost of association studies is reduced to a one-time genotyping expense plus the cost of developing, validating and executing electronic phenotype algorithms; effectively, a queryable record of a diverse set of clinical phenotypes is collected free of charge [40]. Indeed, EHR-derived populations have contributed to recent large meta-analyses [41,42]. Also eliminated is the cost of recruiting patients for each phenotype of study. A recent analysis compared the cost of 115 prior pharmacogenetic studies found in the US National Institutes of Health (NIH) RePORTER system [43] with the estimated costs of 28 EHR-based pharmacogenetic studies [12]. The results showed that the EHR-based approach could reduce study costs by as much as 82% per subject (the median cost per subject per year decreased from US$478 to $96). The study also found that EHR-based studies took a much shorter time than traditional research designs to complete. However, the process of classifying each patient in an EHR population as a case, control or neither for a given phenotype is not easy (discussed in more detail below). Still, for some recent studies, EHR populations for entirely new phenotypes have been derived and classified very rapidly, including for an adverse drug-drug interaction in 20 days [44] and new contributions to meta-analyses in less than a month [42].

The quantity of EHR data provides another significant impetus for their use [45]. Considering that subjects may be clinically complicated - for example, they may have comorbid conditions and be taking multiple medications - a large cohort is essential for further sub-analysis [12]. A recent survey of 456 US biobanks shows that the mean number of specimens per biobank has reached 461,396, and this number is growing rapidly [46].

The availability of longitudinal clinical information in EHRs may also be an asset for genetic research. Certain phenotypes are inherently longitudinal, such as disease complications or progression, survival and drug response [47,48]. Moreover, EHR information can be continuously updated at little cost to the research study. In addition, the inclusion of longitudinal EHR data may lead to more accurate phenotype algorithms [39,49,50]. For example, in one study, differentiating between Crohn’s disease and ulcerative colitis was improved through longitudinal information [51].

## Electronic health record initiatives, projects and workgroups

Beginning in the early 2000s, a number of efforts, networks and collaborations have been repurposing EHR data for genetic research in the United States and beyond. These include the Electronic Medical Records and Genomics (eMERGE) network, national biobanks such as the UK Biobank and China Kadoorie Biobank (CKB), and other efforts such as the Million Veterans Project (MVP) [52] and the Kaiser Permanente Research Program on Genes, Environment, and Health (RPGEH) [53]. These are summarized in Table 1.

The eMERGE network is a pioneering consortium funded by the National Human Genome Research Institute (NHGRI). It initially included five medical research biobanks in 2007 (the Group Health Research Institute, Marshfield Clinic, Mayo Clinic, Northwestern University and Vanderbilt University) and was expanded to nine sites in 2011/2012 (the four new members were Boston Children’s Hospital/Cincinnati Children’s Hospital Medical Center, Children’s Hospital of Philadelphia, Geisinger Health System and Mount Sinai). The primary goal of the eMERGE network is to develop methods and best practices for the utilization of EHRs for genetic research [54,55]. In the past seven years, the eMERGE network has made a significant contribution to the field by demonstrating that data captured through routine clinical care are sufficient to identify various phenotypes for large-scale, high-throughput genetic research. To date, more than 30 electronic phenotype definitions have been created, validated and implemented throughout the network, and the results of genetic replications have been published [36,56-58]. The ‘best practice’ learned from eMERGE is an iterative paradigm of algorithm design followed by physician review of cases and controls in a block-randomized fashion [36].

Pharmacogenomic Discovery and Replication in Very Large Patient Populations (PGPop) [59] is a collaborative research resource of the Pharmacogenomics Research Network (PGRN). Institutions that are part of PGPop investigate drug-response phenotypes through deployment, validation and genetic testing of EHR-linked biobank data. In addition, Kaiser Permanente and the US Department of Veterans Affairs (VA) have launched biobank programs by collecting specimens from their membership populations. Kaiser Permanente started collecting data in 2009, and 200,000 members have now donated their biological samples from the three Kaiser regions (Georgia, Northern California and Oregon). The MVP was initiated by the VA in 2011. Its goal is to enroll one million volunteers and use their clinical and genetic data to improve healthcare for veterans. DNA samples from both biobanks can be linked to EHRs and researchers are allowed to access and use them. EHR biobanks such as MVP, BioVU and BioMe at Mount Sinai [52] include racially and ethnically diverse populations, which could be valuable for future studies of minority groups.

Many European countries have the unique advantages of centralized healthcare systems with long histories of extant data. deCODE [60] and the UK Biobank [61] are two notable European biobanks that have leveraged EHR and insurance claims data. deCODE, a commercial population-based biobank founded in 1996 in Iceland, has been used to investigate the genetics of many common diseases and traits. So far the company has isolated genes thought to be involved in several diseases, such as gout [62], cardiovascular disease [63], cancer [64] and schizophrenia [65]. deCODE is distinct from other biobanks because of the relative genetic homogeneity of the Icelandic population. The clear ‘founder effects’ facilitate the identification of disease genetic etiology. Another unique characteristic of deCODE is that the DNA samples can be linked to their genealogies [66]. Thus, deCODE allows study of the impact of evolutionary factors in human diseases.

The UK Biobank was started in 2007. It collected more than 500,000 volunteers aged from 40 to 69 years and has the ability to request follow-up information. Basic information about participants is obtained through a questionnaire and an interview. Information about clinical visits and issued prescriptions are transferred from the centralized UK National Health Service. The recruitment process was completed in 2010.

Like the eMERGE network, the Nordic Biobank Network is a European collaborative genetics project. It connects several population-based biobanks in the Nordic countries, including Sweden, Finland, Norway, Estonia, Denmark, Iceland and the Faroe Islands. These biobanks contain health information from 25 million inhabitants, including 4 million DNA samples, 100,000 malignant neoplasm samples [67] and 17 million users’ prescription data [68]. Researchers are able to work together to achieve common results and strengthen genetic research.

In East Asia, the CKB aims to explore the complex interplay between genes and environmental factors on the risks of common chronic diseases [69]. Instead of using complete EHRs, the project linked to the national health insurance system and collected abstract outcome data, such as cause-specific mortality, morbidity for a few major diseases and any episode of hospitalization. The BioBank Japan Project also maintains a bio-repository of blood and tissue samples from 300,000 citizens. Its major research focuses are on cancers, diabetes, rheumatoid arthritis and a few common diseases [70,71].

Since EHRs are not fundamentally designed for cross-population queries, the desire to repurpose EHR data for this use has led to the development of research data warehouses. One of the most notable has been Informatics for Integrating Biology and the Bedside (i2b2), an NIH-funded National Center for Biomedical Computing with a primary mission to provide researchers with informatics tools to leverage EHRs for clinical and genetic research [72]. i2b2 developed a scalable computational framework and graphical user interface to allow researchers to query and explore EHR data to create research cohorts. The software it offers can be used for phenotyping from EHRs while preserving patient privacy through a query tool interface. Since 2008, i2b2 has also held annual NLP competitions focused on extracting meaningful computable results from clinical narrative text. Previous challenges included identifying obesity co-morbidities, extracting medication data, identifying smoking status, resolving text co-references (that is, finding all expressions that refer to the same entity in a text; for example, '*The patient* is a 76-year-old lady who has had multiple recurrences of a mandibular mass. *She* also suffers from hypertension, gout, and diabetes mellitus.'), and identifying temporal relationships from text mentions of clinical events (for example, 'the hemorrhage began a week after starting warfarin') [73]. Extraction of information about medications and identification of smoking status have proven particularly valuable to electronic phenotyping [74].

## Genomic replication and discovery using electronic health record data

**Figure 3:**
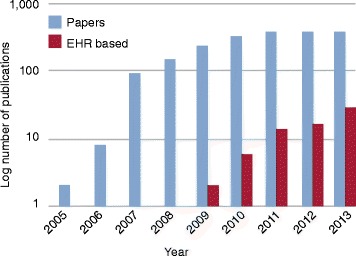
The numbers of GWAS papers and EHR-based genetic studies per year. The horizontal axis represents time. The vertical axis is the log of the number of publications. Data source: National Human Genome Research Institute GWAS Catalog and PubMed.

Below, we review some examples of genetic studies into complex diseases and traits, and drug responses, as well as disease-agnostic approaches such as phenome-wide association studies (PheWASs). The selection of examples is not intended to be comprehensive but instead to provide a sample of the breadth of phenotypes studied and the chronology of EHR exploration for genetic research. Additional file 1 presents a timeline of major milestones in the development of EHR-derived genetic research. The number of publications using EHR-derived biobank samples for genomic research has been rapidly growing in recent years, although it is clearly still dwarfed by non-EHR studies (Figure 3).

### Complex diseases

The first study using EHR data in combination with DNA samples was in 2008. Wood and colleagues enrolled a cohort from patients presenting at a bariatric surgery clinic, collected DNA samples, and then extracted phenotypes from EHRs and tried to replicate two known SNPs associated with coronary heart disease and type 2 diabetes mellitus (T2DM) [75]. They used the International Classification of Diseases, Ninth Revision (ICD-9) codes to define their phenotypes. However, neither of the two SNPs replicated, potentially due to insufficient accuracy of diagnosis codes or the small sample size (709 individuals). In 2010, Ritchie and coworkers applied a more complex phenotyping strategy using a combination of diagnosis codes, procedural codes, laboratory values and clinical notes to define phenotype algorithms for five common diseases: atrial fibrillation, Crohn’s disease, multiple sclerosis, rheumatoid arthritis and T2DM [51]. Physicians reviewed the electronic medical records to determine whether the cases and controls identified by the algorithms were correctly labeled. Of note, algorithms were used to identify both cases and controls, such that many individuals were neither cases nor controls due to insufficient information or potentially overlapping diseases. Their manual chart review showed that the positive predictive values (PPVs) of algorithms reached 95% or better. In the following analysis, they replicated at least one previously reported association for each of the diseases. Another group conducted a replication study on rheumatoid arthritis [76]. They also used both structured and unstructured EHR data to define the rheumatoid arthritis phenotype. Their results showed that the odds ratios and aggregate genetic risk score (GRS) of known rheumatoid arthritis risk alleles were nearly identical to those reported from a previous meta-analysis of multiple traditionally collected cohorts.

Several projects have discovered new genetic associations using EHR-linked DNA biobanks for genetic discovery [77]. For example, eMERGE investigators reported common variants near the forkhead family gene *FOXE1* associated with hypothyroidism in European-Americans [50]. Chen and colleagues leveraged the absolute lymphocyte count from clinical data to identify 53 maturation/aging-related genes [78]. Other novel associations were found using GWASs of erythrocyte sedimentation rate [79], red blood cell counts [80] and varicella zoster virus infection [81], among others [77].

Since EHRs became available for research, investigators have studied the portability of EHR-based phenotype definitions. Many phenotype definitions of complex diseases, such as hypothyroidism [50], cardiovascular diseases [82-84], T2DM [57] and rheumatoid arthritis [56,85], have been deployed and validated across multiple institutions. EHR-derived phenotypes appear to be generally portable and more accurate than previous designs using just administrative data, and are therefore gaining more widespread acceptance for clinical and genetic research [13,86]. Now, researchers are able to study phenotypes at different levels of detail - for example, drug-dose response [48,87,88] versus longitudinal analyses [89,90]. Many of these algorithms from eMERGE and other institutions have been shared on the Phenotype KnowledgeBase [40].

Studies combining genotyping and phenotyping not only proved the utility of linking EHR data with biospecimens for genetic studies but also suggested that electronic phenotyping is not as straightforward as simply querying patient data for diagnosis codes. Challenges in defining phenotypes still exist, and at present computational methods to share complicated phenotypes across EHR systems or institutions do not exist. Thus, each site must use local informatics personnel to deploy the algorithm, and manual chart review is required for validation. Indeed, manual curation of all records may be required for some phenotypes if they have low PPVs [48,91]. Successful phenotyping may require the collaboration of clinicians, informaticians and other domain experts to develop a validated algorithm.

### Pharmacogenomics

Pharmacogenomics seeks to identify the genetic underpinnings affecting an individual’s response to drugs. However, partially owing to the difficulty of obtaining cohorts with drug-response data, pharmacogenomics has not been thoroughly studied. We reviewed the 1,920 studies in the NHGRI GWAS catalog as of September 2014 and noted that only 7% of them include drug-response phenotypes, with most of these studies focusing on the efficacy of warfarin, chemotherapy and psychiatric medications. Thus, pharmacogenomics may be a ripe area for research using EHR data [35]. Indeed, EHR data have already been used to successfully replicate associations with clopidogrel, warfarin and tacrolimus. Variants in the membrane-transporter-encoding gene *ABCB1* and the cytochrome P450 gene *CYP2C19* were associated with recurrent cardiac events during clopidogrel therapy in a real practice setting using EHR data [48]. Birdwell and coworkers confirmed the association of tacrolimus blood concentration to dose ratio with the *CYP3A5* gene variant rs776746 using transplant patients and their EHR data for medication doses and tacrolimus levels [92]. Ramirez and colleagues investigated the associations between steady-state warfarin dose and European-American or African-American ancestry using EHRs [88]. Integration of an expanded set of genetic variants into a warfarin pharmacogenomic algorithm improved dose prediction, reducing the prediction error by 23% in European-Americans and by 7.5% in African-Americans when compared to clinical algorithms. A later study of warfarin-treated individuals demonstrated that the CYP2C9*3 variant conferred a twofold increased risk of warfarin-related bleeding events after the warfarin initiation period [93].

Besides the replication and expansion of pharmacogenetics findings, EHRs have been used to discover novel pharmacogenetics-related phenotypes. For example, a study group from the Marshfield Clinic used their biobank to identify an estrogen receptor genotype associated with thromboembolism during tamoxifen exposure [94]. Another study generated dose–response curves for atorvastatin and simvastatin to test both potency and efficacy of the drugs for association with 144 preselected SNPs [87]. They identified a pharmacodynamic variant (in the transcriptional regulator *PRDM16*) associated with statin efficacy and several loci associated with potency. EHRs have also contributed to a meta-analysis of statin reduction of low-density lipoprotein (LDL) cholesterol levels [42]. Furthermore, EHR data have uncovered variants in the G-protein-coupled receptor gene *TDAG8* (also known as *GPR68*) associated with heparin-induced thrombocytopenia, a rare but severe adverse reaction to heparin anticoagulant therapy [95].

### Phenome-wide approaches

By virtue of serving as the record of an individual’s clinical history, EHRs represent an agnostic collection of phenotypes driven by the reasons for a patient to seek healthcare. As such, EHRs enable a new class of research that looks at many different diseases simultaneously. For example, Rzhetsky and colleagues used billing codes from the EHRs of 1.5 million patients to analyze disease co-occurrence in 161 conditions, demonstrating that autism, bipolar disorder and schizophrenia likely share significant genetic architecture [96]. This inference was later validated using GWAS data on the three diseases [97]. Another study of autism spectrum disorders analyzed the longitudinal diagnosis codes of 13,740 individuals and observed three distinct new patterns of medical trajectories [89]. The findings confirmed the value of longitudinal EHR data and implied various genetic etiologies for the disease.

PheWASs provide a systematic scan of clinical phenotypes associated with a target genetic variant. As such, a PheWAS can be considered as a ‘reverse GWAS’. In a PheWAS in 2010, groups of diagnosis codes were used as phenotypes to replicate previously known gene-disease associations for seven common diseases. Associations of four diseases were successfully replicated, including multiple sclerosis, rheumatoid arthritis, Crohn’s disease and ischemic heart disease [98]. A more recent PheWAS of 3,141 variants testing 751 SNP-phenotype associations previously discovered through a GWAS replicated 210 of them, including 66% of known associations with adequate sample size to be tested for in the cohort. This study also identified 63 new associations, some of which represent true pleiotropy, in which the genetic variant is associated with multiple distinct phenotypes [99]. Hebbring and coworkers replicated a novel PheWAS finding of an association between the human leukocyte antigen HLA-DRB1*1501 variant and erythematous rashes in the Marshfield Clinic biobank [100] and have subsequently leveraged this cohort to study functional variants across the genome [101]. Cronin and team used this approach to identify an association between obesity-associated *FTO* variants and fibrocystic breast disease [102]. Namjou and colleagues applied the same approach to European-origin pediatric cohorts and discovered genetic links between the phospholipase C-like 1 gene *PLCL1* and speech language development, and between the interleukin gene cluster *IL5-IL13* and eosinophilic esophagitis [103]. A study by Shameer and team revealed that variants associated with the number of circulating platelets and mean platelet volume have pleiotropic associations with myocardial infarction, autoimmune and hematologic disorders [104]. The PheWAS approach has also been used in observational cohorts [105]. These independent validations confirmed the feasibility of PheWASs for genetic research.

## Challenges of repurposing electronic health record data for genetic research

EHRs are primarily designed for clinical care, not research. As a result, reuse of EHRs for research purposes poses certain challenges. These challenges result from imperfections in the EHR data themselves and challenges in ‘understanding’ the EHR data for phenotype abstraction.

EHRs derive from selected populations and their data contain biases [34,45,106]; in particular, they are biased toward sick individuals. In addition, a study of longitudinal Medicare claims data showed substantial differences in diagnostic practices across various US regions [107]. As a consequence, when EHR data are repurposed for genetic research, biases in the phenotyping output should be considered and evaluated. Controls may also contain biases based on the reason the population was selected, the EHR from which they were derived, or insufficient data within the EHR to rule out them having the disease. For example, consider a patient seen only for an orthopedic concern, such as a fracture, and its follow-up; the individual may have multiple elevated blood pressure readings due to pain (and appear to be a case for hypertension) and never receive glucose screening to rule out diabetes (and thus may seem to be a candidate for a control for diabetes). Novel approaches, statistical or informatics-based, are needed to handle observation biases of data in the EHR. One recent study found improved association results by matching controls to cases based on density of EHR content [108].

Undoubtedly, results of phenotyping would be more accurate if all EHR data for every patient were available. However, clinical data are often fragmented across healthcare systems as patients visit multiple healthcare centers, change insurance, and move. The ability to exchange EHR data is limited [109]. A recent retrospective observational study indicated that, of the nearly 3.7 million patients who sought treatment in acute care settings in Massachusetts, over 30% visited more than one hospital and 1% visited five or more hospitals [110]. Similar findings were reported in another cross-sectional survey conducted in 32 primary care clinics in Colorado, which suggested that missing information in clinical settings is common and multifaceted [111]. Incomplete EHR data may adversely affect phenotyping results. A study evaluating the eMERGE T2DM algorithm [57,98] found that using EHR data from two medical centers in Minnesota had better predictive power than using data from one medical center alone [112]. A follow-up study found that phenotype accuracy improved as the timeframe of available EHR data was increased from one to ten years [49].

Another issue limiting repurposing EHRs for research is EHR accuracy. Inaccuracy in an EHR may be introduced at any time during a clinical visit; billing accuracy is not always a high priority for busy clinicians. Common sources of inaccuracy include the amount and quality of information available, communication between patients and clinicians, professional knowledge and experience with the illness, unintentional errors (for example, misspecification, use of medical abbreviations), and, occasionally, intentional errors (for example, upcoding diagnoses for higher restitution) [113]. Additionally, EHRs can record and store data in different ways. For example, ‘weight’ and ‘height’ may be recorded and stored within an EHR system in different units (for example, kilograms, grams and pounds for weight), which can lead to false body mass indices [86]. Acronyms may have multiple meanings, such as ‘RA’ (rheumatoid arthritis, right atrium, room air or right arm) and ‘PD’ (Parkinson’s disease or personality disorder), and are frequently found in clinical notes [114]. In addition, a failed laboratory test or a contaminated blood sample may return a physiologically unlikely value, such as an LDL over 10,000 mmol/l. These inaccuracies do not typically misdirect a provider’s diagnosis or treatment as clinicians can easily discern any mistakes or decode acronyms based on the available context and their medical knowledge. However, the lack of such knowledge makes it difficult for a computer to detect or determine the correct information, thus resulting in phenotyping false positives.

EHR data are highly complex and include both structured and unstructured information that must be woven together to create a phenotype algorithm [109,115]. In recent years, considerable NLP efforts have been devoted to promoting information extraction from clinical notes, resulting in many publicly available or home grown NLP systems, such as cTAKES [116], MedLEE [117] and KMCI [118]. However, subtle relationships hidden in notes remain difficult to extract due to the complexity of the language used and the lack of explicit semantic resources describing the relationships between clinical concepts [119,120]. A combination of deeper syntactic analysis and domain knowledge stored in formal ontologies would be a promising future direction.

Another challenge to broad use of EHR data is that they contain protected health information. Many EHR-linked biobanks have been collected under consent models that assume protection of the individual’s identity. Some EHRs include the consent and information necessary to re-contact individuals [121,122] while others do not [123]. Given publicly available resources, researchers have shown that removal of the specific identifiers mandated by the US Health Insurance Portability and Accountability Act (HIPAA) is insufficient to protect against re-identification [124,125]. For this reason, most EHR-linked biobanks are protected with access policies, and result sets that are shared publicly (for example, with dbGaP) are analyzed for re-identification risk. Additionally, the NIH’s Genomic Data Sharing (GDS) policy [126], which went into effect on 25 January 2015, requires individuals to consent to broad data sharing of their DNA (in a manner compliant with HIPAA Safe Harbor). This policy made untenable some existing opt-out consent models for future federal studies, such as that employed in the Vanderbilt BioVU biobank [123]. As a result, BioVU, as one example, has transitioned to an opt-in consent model for future studies that explicitly consents for data sharing. However, the GDS policy states that samples collected before 25 January 2015 in cohorts not explicitly consented for sharing (such as BioVU) can still be used in future NIH studies.

## Conclusions and future directions

Accumulated studies suggest that EHRs offer potential efficiencies in addressing the temporal and economic challenges of traditional genetic research. Ample EHR data may enable the extraction of more reliable and fine-grained phenotypes. The number of EHR studies is growing. To date, EHR biobanks with extant genetic data are relatively small compared to the largest meta-analyses. A near-term future expectation, however, is that millions of patients for whom EHR data are available will also have available genetic data through efforts such as eMERGE and MVP, and national biobanks such as the UK Biobank, CKB and Qatar Biobank. These efforts will make EHR biobanks an important and growing resource for data discovery and replication. Indeed, effective use of EHR data will likely play an important role in the US Precision Medicine project announced by President Obama in his State of the Union address on 20 January 2015.

One of the key lessons that we have learned from previous experience is that work is needed to define phenotypes accurately using EHR data. Accurate phenotypes have become a rate-limiting step for EHR-based genetic research, and the process of accurately defining them often requires interactions between subject matter experts and informaticians in an iterative process of refinement [127]. The Health Information Technology for Economic and Clinical Health (HITECH) Act, enacted as part of the American Recovery and Reinvestment Act of 2009, may increase the availability of EHRs for genetic research. Owing to the Meaningful Use Regulations, which are particularly aimed at increasing the capability for clinical information exchange, large-scale adoption of these certified EHR technologies and agreed standards for interoperability will accelerate the exchange of phenotypic and genetic data across various systems, thereby forming a more powerful ‘EHR cloud’ than ever before [128]. However, there is no current standard for applying automated, fully computable and transportable execution of phenotype algorithms to a diverse set of EHR systems and sites. The closest current effort is perhaps the Quality Data Model [129]; however, this specification at present does not allow for depth of NLP or complex methods such as machine learning, seen in some phenotyping algorithms [130].

Unfortunately, many data in clinical records are still not computable. New knowledge resources and applications of structured medical terminologies may improve the ‘computability’ of future EHRs. Pioneering work includes standardized vocabularies such as Systematized Nomenclature of Medicine - Clinical Terms (SNOMED-CT) for representing clinical concepts such as diseases and clinical traits, RxNorm for medications, and the Unified Medical Language System (UMLS) to link >100 disparate vocabularies together. Some of these vocabularies offer predefined semantic relationships that can be leveraged in future applications. For example, SNOMED-CT includes links between its nearly 400,000 concepts with an extensive hierarchical structure, along with other semantic relationships [131]. In this way, a computer can computationally deduce that the concept ‘viral pneumonia’ is an ‘infective pneumonia (disorder)’, which has a ‘causative agent’ relationship with the concept ‘virus’ and a ‘finding site’ relationship with the concept ‘lung’. Some efforts, such as openEHR [132] and clinical element model (CEM) [133], have published specifications to define detailed clinical data. The implementation of formal representations of EHR data may improve automatic phenotyping performance because computers may ‘understand’ the meaning across clinical data based on pre-defined semantics.

Fully leveraging the potential of EHRs often requires not only knowledge within a terminology but also of the semantic relationships between concepts across terminological systems. For example, drugs are typically used for disease management (indications) and they may also cause problems (side effects). The ICD-9 and RxNorm are used to represent diseases and drugs, respectively, but neither of them maintains the knowledge of indications and side effects. Although terminological systems such as the UMLS are often used to bridge terminologies, the relationship between concepts across terminologies remains suboptimal. Some groups have created *ad hoc* mapping between concepts across terminologies. This manual approach is time consuming and faces significant challenges due to the disparity of coverage and granularity between terminologies [134-136]. We and others have investigated one particular relationship (for example, indication) at a time and leveraged available resources to identify concepts from different terminologies applicable to this relationship. This approach has led to several previously unavailable resources, such as SIDER [137] and MEDI [138-140]. SIDER offers information about drugs and their corresponding side effects. MEDI provides computable knowledge about drugs (represented by RxNorm concepts) and their indications (represented by the ICD-9 or UMLS Concept Unique Identifiers). These knowledge bases have proven beneficial to many other studies - for example, in drug discovery [141] and clinical information extraction [142]. EHR-based genetic research requires knowledge from basic science, clinical practice and informatics. Anticipation of increased use of ontologies within clinical information systems and biological resources from various domain terminologies - for example, Gene Ontology, SNOMED-CT and ICD-9 - would facilitate conjoined knowledge bases to accelerate research and cross-talk between biological research and clinical care.

Advanced tools for unstructured EHR data analysis not limited to narrative notes will improve the quality and detail of future phenotypes extracted from the EHR. However, a number of challenges still exist, such as disambiguation of acronyms and interpretation of clinical meaning across a number of sentences. Other unstructured data - for example, radiology images and waveform data - may be key to diagnosis in routine practice, such as using chest X-rays to rule out pneumonia and electrocardiography for myocardial infarction. Few of these raw data are involved in electronic phenotyping at present. In the future, EHRs may routinely include pictures (of rashes, for example) and radiological data that can be readily reprocessed with imaging algorithms, and abundant sensor data such as telemetry or mobile health technologies will be available - providing another deep resource that would be costly to obtain outside of clinical care.

In addition, new models will be needed to handle many-to-many gene-disease analysis. For example, researchers frequently observe that certain diseases (for example, diabetes and hypertension) co-occur in individuals, suggesting a possible many-to-many association between genetic variations and multiple disorders. Network analyses may help untangle such complex relationships.

The ultimate utility of genetic discovery will be tested through its implementation in clinical practice. The challenge of incorporating genetic data and implementing decision support has been discussed elsewhere [128]. EHRs need to be adapted to handle new and large classes of information, new standards must be created and adopted, and decision support should be refined to ensure that genetic findings are seamlessly integrated into clinical workflow. A few medical centers have already incorporated genetic information into routine care [143-145]. These centers have shown that genomic data can be used to tailor prescribing decisions to target therapies better [146,147] and to avoid serious drug adverse events [148,149], which are often impossible to predict without using genetics. Acceleration of the adoption of genomic medicine is also the goal of NHGRI’s IGNITE network, which includes a wide array of underserved, community, VA and military medical centers [150]. In these ways, NIH director Francis Collins’ 2009 vision of a genomic treatment plan for a patient being 'simply a click of the mouse' away is already being realized for some conditions [151].

## Additional file

Additional file 1:
**Timeline of genetic and electronic health record-based research.** A timeline of major milestones in the development of EHR-derived genetic research.

## Abbreviations

CKB: China Kadoorie Biobank
CPT: Current Procedural Terminology
EHR: electronic health record
eMERGE: The Electronic Medical Records and Genomics network
GDS: Genomic Data Sharing
GRS: genetic risk score
GWAS: genome-wide association study
HIPAA: Health Insurance Portability and Accountability Act
HITECH: Health Information Technology for Economic and Clinical Health
i2b2: Informatics for Integrating Biology and the Bedside
ICD-9: International Classification of Diseases, Ninth Revision
KP: Kaiser Permanente
MVP: Million Veteran Program
NIH: National Institutes of Health
NHGRI: National Human Genome Research Institute
NLP: natural language processing
PGRN: Pharmacogenomic Reseach Network
PGPop: Pharmacogenomic Discovery and Replication in Very Large Patient Populations
PheWAS: phenome-wide association study
PPV: positive predictive value
RPGEH: Research Program on Genes, Environment, and Health
SNOMED-CT: Systematized Nomenclature of Medicine - Clinical Terms
SNP: single nucleotide polymorphism
T2DM: type 2 diabetes mellitus
UMLS: Unified Medical Language System
VA: US Department of Veterans Affairs
