# Supplementary figures and images for: Extracting research-quality phenotypes from electronic health records to support precision medicine
Source: Genome Med. 2015 Apr 30;7(1):41. doi: 10.1186/s13073-015-0166-y (PMC4416392; doi:10.1186/s13073-015-0166-y)

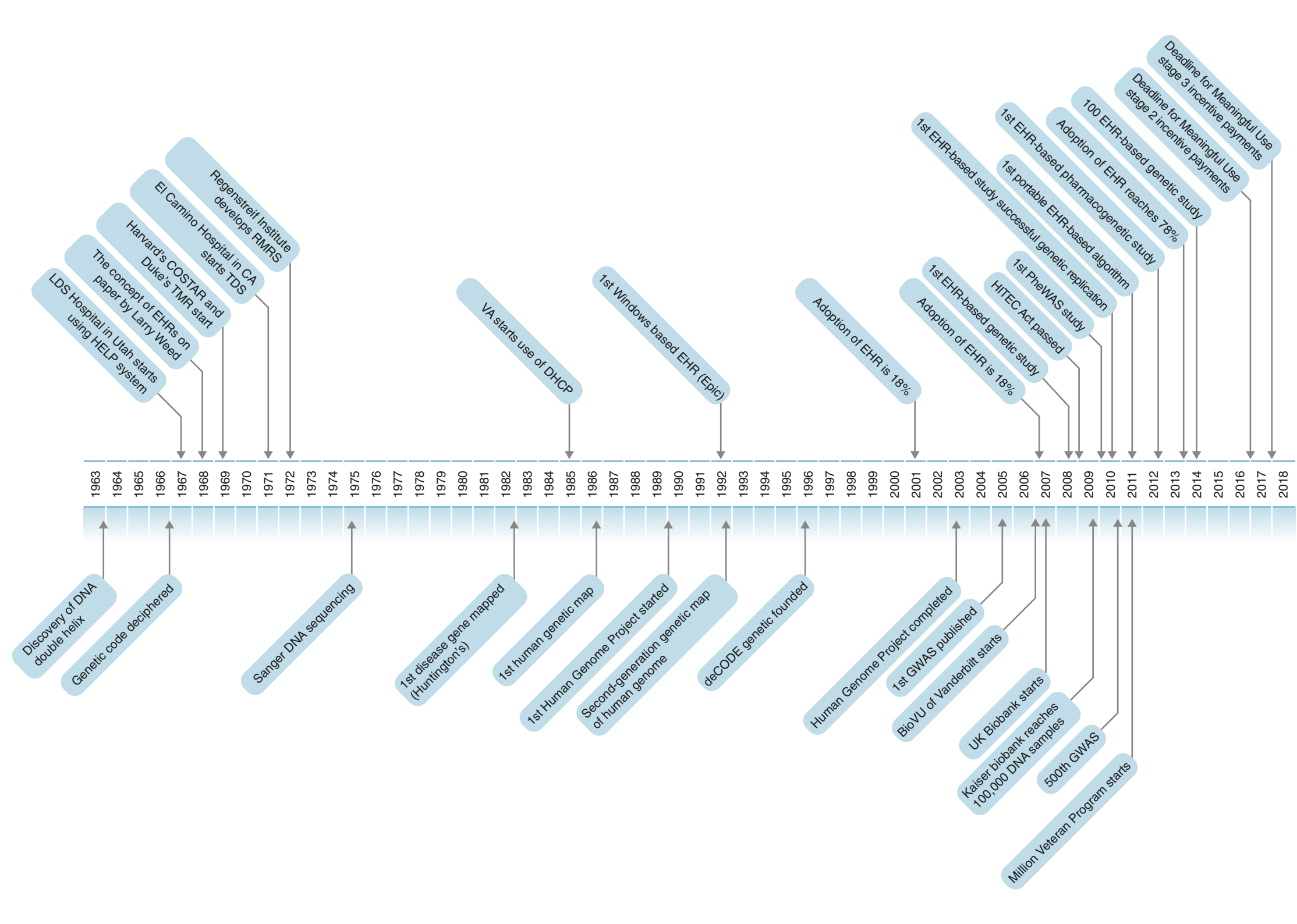

Supplement: Additional file 1: — Timeline of genetic and electronic health record-based research. A timeline of major milestones in the development of EHR-derived genetic research. [file 13073_2015_166_MOESM1_ESM.pdf]
